# *IFITM3*, *FURIN*, *ACE1*, and *TNF-α* Genetic Association With COVID-19 Outcomes: Systematic Review and Meta-Analysis

**DOI:** 10.3389/fgene.2022.775246

**Published:** 2022-04-01

**Authors:** João Locke Ferreira de Araújo, Diego Menezes, Renato Santana de Aguiar, Renan Pedra de Souza

**Affiliations:** Grupo de Pesquisa em Bioestatística e Epidemiologia Molecular, Laboratório de Biologia Integrativa, Programa de Pós Graduação em Genética, Departamento de Genética, Ecologia e Evolução, Instituto de Ciências Biológicas, Universidade Federal de Minas Gerais, Belo Horizonte, Brazil

**Keywords:** polymorphism, genetic association study, candidate genes, transposable elements, biomarkers, host genetics

## Abstract

Human polymorphisms may contribute to SARS-CoV-2 infection susceptibility and COVID-19 outcomes (asymptomatic presentation, severe COVID-19, death). We aimed to evaluate the association of *IFITM3*, *FURIN*, *ACE1*, and *TNF-α* genetic variants with both phenotypes using meta-analysis. The bibliographic search was conducted on the PubMed and Scielo databases covering reports published until February 8, 2022. Two independent researchers examined the study quality using the Q-Genie tool. Using the Mantel–Haenszel weighted means method, odds ratios were combined under both fixed- and random-effect models. Twenty-seven studies were included in the systematic review (five with *IFITM3*, two with *Furin*, three with *TNF-α*, and 17 with *ACE1*) and 22 in the meta-analysis (*IFITM3 n* = 3, *TNF-α*, and *ACE1 n* = 16). Meta-analysis indicated no association of 1) *ACE1* rs4646994 and susceptibility, 2) *ACE1* rs4646994 and asymptomatic COVID-19, 3) *IFITM3* rs12252 and ICU hospitalization, and 4) *TNF-α* rs1800629 and death. On the other hand, significant results were found for *ACE1* rs4646994 association with COVID-19 severity (11 studies, 692 severe cases, and 1,433 nonsevere controls). The *ACE1* rs4646994 deletion allele showed increased odds for severe manifestation (OR: 1.45; 95% CI: 1.26–1.66). The homozygous deletion was a risk factor (OR: 1.49, 95% CI: 1.22–1.83), while homozygous insertion presented a protective effect (OR: 0.57, 95% CI: 0.45–0.74). Further reports are needed to verify this effect on populations with different ethnic backgrounds.

**Systematic Review Registration**: https://www.crd.york.ac.uk/prosperodisplay_record.php?ID=CRD42021268578, identifier CRD42021268578

## Introduction

Coronavirus disease 2019 (COVID-19) clinical presentation is heterogeneous, ranging from entirely asymptomatic up to severe cases and death. Another level of heterogeneity is observed regarding persistent symptoms: one study has estimated that the median proportion of individuals who experienced at least one persistent symptom was 73% (Nasserie et al., 2021). Uncovering biomarkers linking patients with distinct prognosis subgroups would be beneficial. Different strategies have been employed to uncover molecular markers predicting odds for better prognosis and severe acute respiratory syndrome coronavirus 2 (SARS-CoV-2) infection susceptibility. Proteins, lipids, and metabolites have already been examined (Praissman and Wells, 2021; Samprathi and Jayashree, 2021). Genetic variability has been shown to be a valuable source for biomarker research. COVID-19 prognosis and infection susceptibility are multifactorial traits determined by the complex interaction of environmental factors and multiple genes. Thus, significant single-gene results may lead to substantial predictors such as the C–C chemokine receptor type five (*CCR5*) gene association with HIV susceptibility and prognosis (Liu et al., 2012), or ABO blood type and dengue severity (Hashan et al., 2021).

Genetic association studies can be designed within prespecified genes of interest (candidate gene approach) or with a broader strategy characterizing diversity across large genomic areas (genome-wide association studies, whole exome and genome sequencing). Angiotensin-converting enzyme 2 (*ACE2*), transmembrane serine protease 2 (*TMPRSS2*), human leukocyte antigen (*HLA*), interferon-induced transmembrane protein 3 (*IFITM3*), tumor necrosis factor-alpha (*TNF-α*), *FURIN*, and angiotensin I-converting enzyme (*ACE1*) were the most studied genes using the candidate gene approach in 2020 (Araújo et al., 2021). They all present strong biological plausibility since they act on viral cell entry or human immune response to SARS-CoV-2.

Findings from single association studies must always be considered carefully because of the likelihood of producing spurious outcomes (Sullivan, 2007). Replication is essential before considering using genetic markers in the clinical setting. Although that has been proved hard, inconsistency frequently can be attributed to shortfalls in study design, implementation, and interpretation, with inadequately powered sample groups being of significant concern (Hattersley and McCarthy, 2005). A systematic meta-analytic approach may support estimating population-wide effects of genetic risk factors in human diseases (Ioannidis et al., 2001). The PROSPERO (Moher et al., 2014) database, indicating protocols for systematic reviews, has already been presented for *HLA* (CRD42021251670) (Deb et al., 2022), *ACE2*, and *TMPRSS2* (CRD42021229963) contribution with COVID-19 outcomes. Therefore, we focused our systematic review on *IFITM3*, *FURIN*, *ACE1*, and *TNF-α* genetic variants and their association with COVID-19 susceptibility and prognosis to reduce unnecessary duplication.

*IFITM3* (MIM 605579; 11p15.5) is a protein-coding gene that disturbs cell entry by inhibiting viral fusion with cholesterol-depleted endosomes (Amini-Bavil-Olyaee et al., 2013); a mechanism also described during SARS-CoV-2 infection (Prelli Bozzo et al., 2021). The *IFITM3* rs12252 polymorphism has been associated with influenza severity (Prabhu et al., 2018). The *TNF* (MIM 191160; 6p21.33) gene encodes a multifunctional proinflammatory cytokine. Although TNF-α is not as relevant as interleukin-6 on the cytokine storm presented in severe COVID-19 patients (Karki and Kanneganti, 2021), anti-TNF-α drug repositioning for COVID-19 has been proposed (Stebbing et al., 2020). FURIN is coded by the *FURIN* (MIM 136950; 15q26.1) gene. It regulates constitutive exocytic and endocytic pathways and has a central role in SARS-CoV-2 transmission (Peacock et al., 2021). The *ACE1* (MIM 106180; 17q23.3) gene produces a protein related to blood pressure regulation and electrolyte balance, and ACE1/ACE2 balance has been suggested to play a pivotal role in the pathobiology and treatment of COVID-19 (Sriram and Insel, 2020). The *ACE1* rs4646994 variant is a 287-bp Alu repeat insertion/deletion (indel) on intron 16 known to alter ACE-1 levels and influence several clinical traits (Castellon and Hamdi, 2007). Here, we present the result of a systematic review and, whenever possible, a meta-analysis of *IFITM3*, *FURIN*, *ACE1*, and *TNF-α* genetic association with susceptibility to SARS-CoV-2 infection and COVID-19 severity.

## Materials and Methods

The systematic review protocol was submitted to PROSPERO (CRD42021268578). Preferred Reporting Items for Systematic reviews and Meta-analysis (PRISMA) was adopted as a guideline for reporting this systematic review (Page et al., 2021). The study selection was carried out in three phases: identification, screening, and eligibility. Search on the PubMed and Scielo databases led to article identification. The PECO question for prognosis was Participants (P) = subjects with COVID-19, Exposition (E) = minor alleles, Control (C) = major alleles of genetic variants, and Outcomes (O) = COVID-19 severity (asymptomatic or severe presentation); while the PECO question for susceptibility was P = overall population, E = minor alleles, C = major alleles of genetic variants, and Outcomes (O) = COVID-19 positive diagnosis. The bibliographic search included all studies published until February 8, 2022, with no language restriction, using the search arguments listed in Supplementary Material SI. Two independent researchers conducted article screening. Inclusion criteria were primary articles covering genetic association of COVID-19 susceptibility or prognosis with *IFITM3*, *FURIN*, *ACE1*, and *TNF-α* variants, comprising four separate searches. Exclusion criteria were review articles or primary articles evaluating the association of COVID-19 susceptibility or prognosis with other genes.

We assessed study quality using the Q-Genie tool (Sohani et al., 2016) performed by two independent researchers. This instrument contains 11 questions to be marked on a seven-point Likert scale examining several aspects of a genetic association study: scientific basis for the development of the research question, ascertainment of comparison groups (e.g., cases and controls), technical and nontechnical classification of tested genetic variants (e.g., genotyping call rates, blinded experiments), classification of the outcome (e.g., sampling strategy, definition criteria), discussion of sources of bias, appropriateness of sample size, description of planned statistical analyses, statistical methods applied, test of assumptions in the genetic studies (e.g., Hardy–Weinberg equilibrium), and appropriate interpretation of the results. Proposed cutoffs for understanding are ≤35 poor, > 35 moderate, and ≥45 good quality, with the total score ranging from 7 to 77 points.

Meta-analysis was conducted whenever three or more studies were included for the same polymorphism and outcome. We carried out single meta-analyses for each polymorphism considering allelic and genotypic effects (under both allele recessive model assumptions). Heterogeneity between studies was assessed using the chi-square test. We used the *metabin* function coded on *meta* package in R (version 4.1.0) (R Core Team, 2014) to estimate overall odds ratios (ORs) and its 95% confidence interval (CI). Original ORs were combined using the Mantel–Haenszel weighted means method under both fixed- and random-effect models. The significance level was set at 0.05.

## Results

**FIGURE 1:**
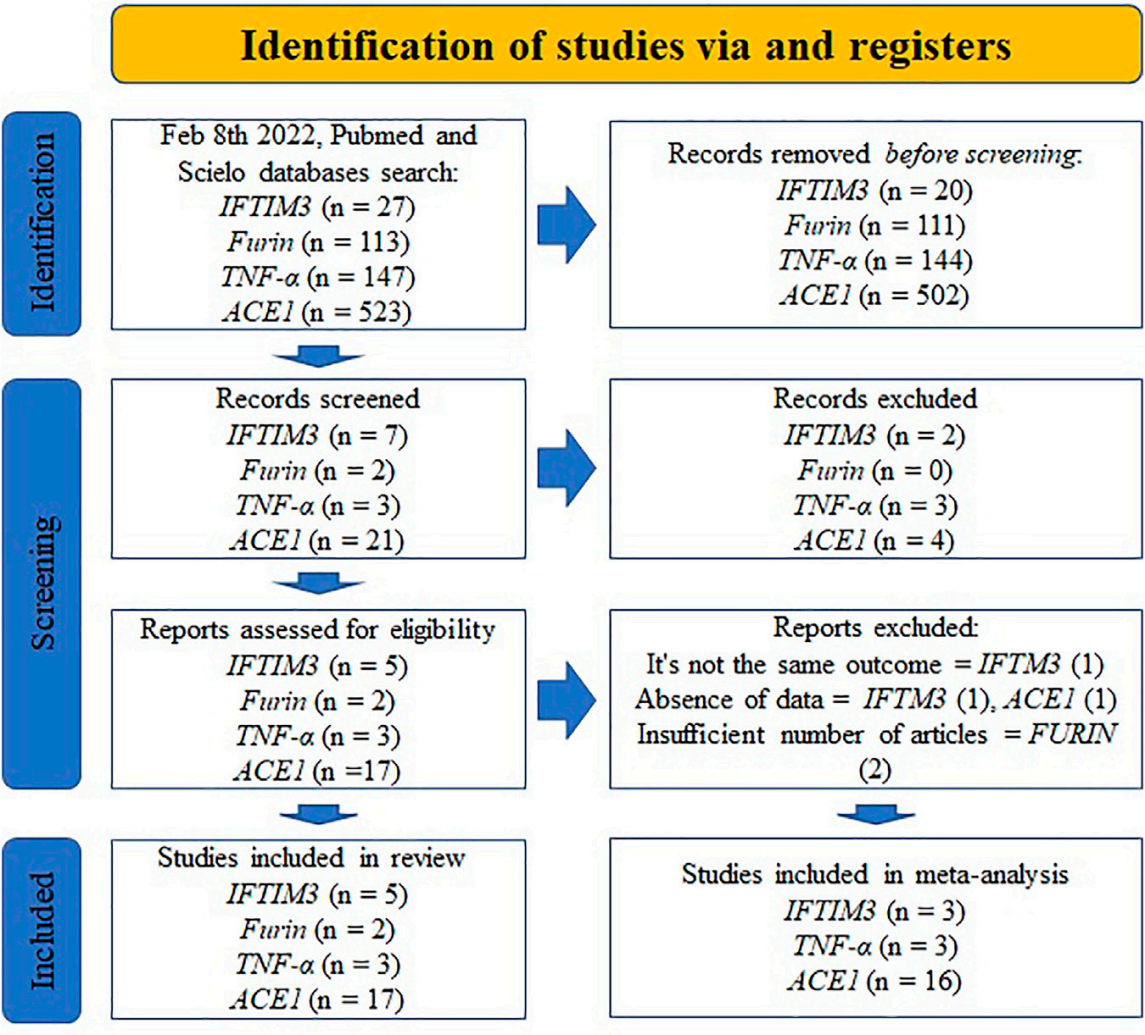
Study selection using Preferred Reporting Items for Systematic reviews and Meta-analysis (PRISMA) guidelines (16).

Twenty-seven studies were included in the systematic review: five with *IFITM3* (Zhang et al., 2020; Alghamdi et al., 2021; Cuesta-Llavona et al., 2021; Gómez et al., 2021; Schönfelder et al., 2021), two with *Furin* (Latini et al., 2020; Torre-Fuentes et al., 2021), three with *TNF-α* (Saleh et al., 2020; Fishchuk et al., 2021; Heidari Nia et al., 2021), and 17 with *ACE1* (Gómez et al., 2020; Aladag et al., 2021; Annunziata et al., 2021; Cafiero et al., 2021; Gunal et al., 2021; Hubacek et al., 2021; Karakaş Çelik et al., 2021; Kouhpayeh et al., 2021; Mir et al., 2021; Möhlendick et al., 2021; Saad et al., 2021; Verma et al., 2021; Akbari et al., 2022; Gong et al., 2022; Mahmood et al., 2022; Papadopoulou et al., 2022). (Figure 1). Inconsistencies in reported frequencies were found in two studies (Gómez et al., 2021; Karakaş Çelik et al., 2021).

All manuscripts but one reached moderate or good quality scores in the Q-Genie analysis (Supplementary Material S1). Among the 11 questions, it is clear that all studies had the worst performance for questions number 5 and 10. While question 5 examines reported information regarding how genotyping was conducted (e.g., blinded experiments, batch effects), question 10 evaluated whether genetic relationships among subjects were tested, and sex and ethnicity were stated.

**FIGURE 2:**
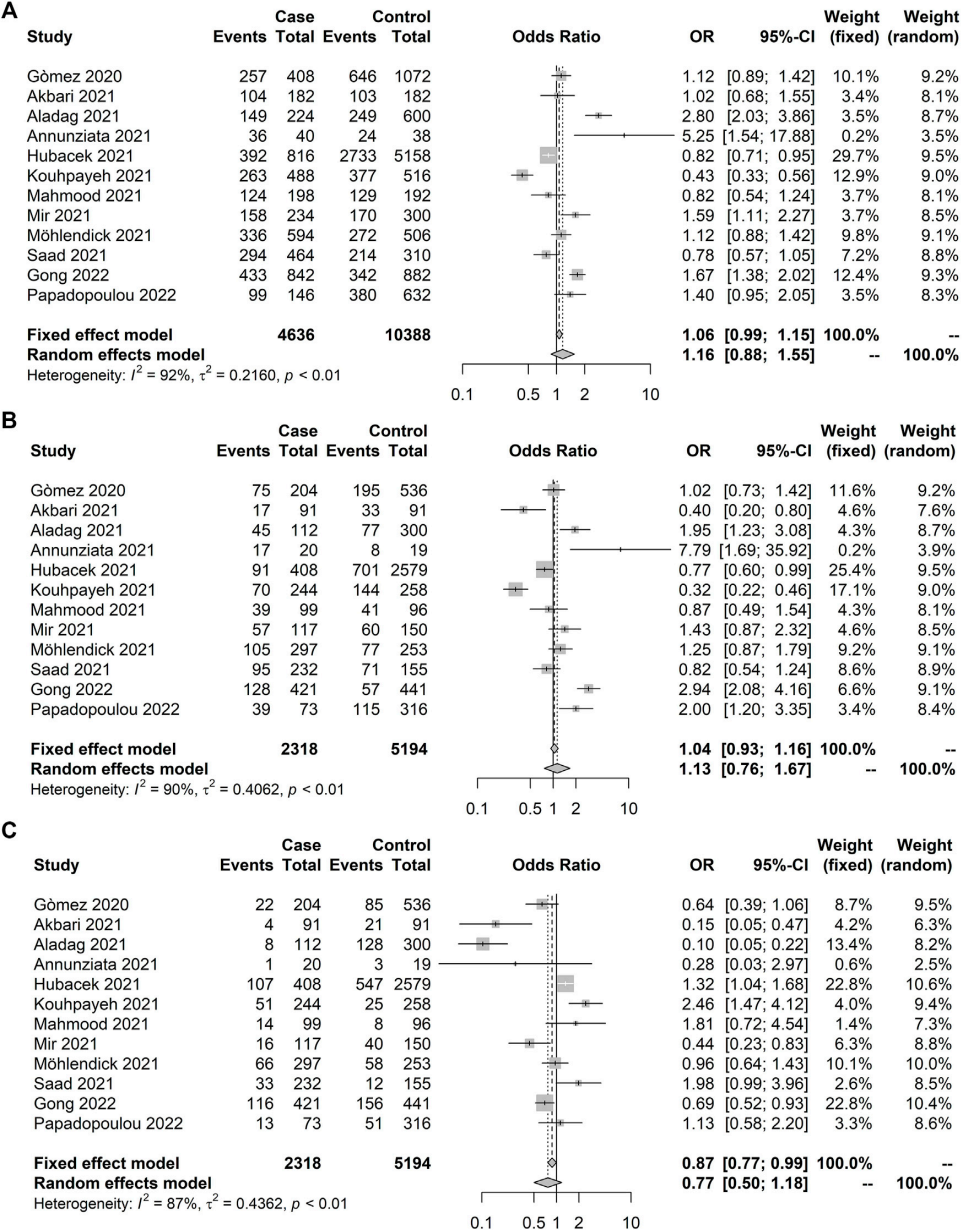
Forest plot illustrating *ACE1* rs4646994 (Alu 287 pb) association with coronavirus disease 2019 (COVID-19) susceptibility. No significant results were observed. Case and control definitions are presented in Table 1. **(A)** C allele association. **(B)** C recessive model. **(C)** T recessive model.

**FIGURE 3:**
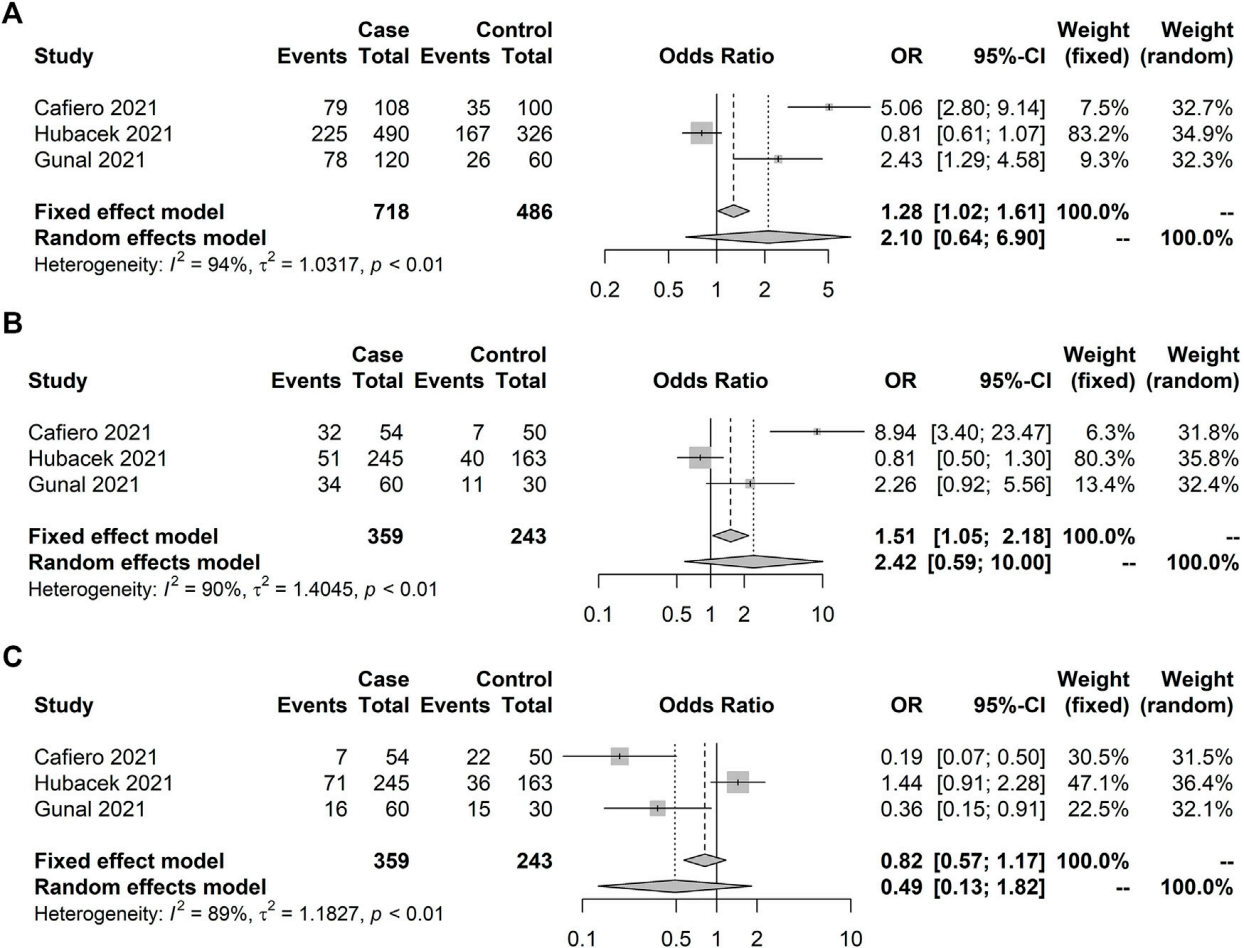
Forest plot illustrating *ACE1* rs4646994 (Alu 287 pb) association with symptom presence (asymptomatic × symptomatic). No significant allelic and genotypic effects were observed under the random model. Case and control definitions are presented in Table 2. **(A)** D-allele model. **(B)** D recessive model. **(C)** I recessive model.

Five meta-analyses were carried out, including 22 studies evaluating three genes (*IFITM3 n* = 3, *TNF-α* n = 3, and *ACE1 n* = 16). Twelve studies, including 2,318 control subjects and 5,194 COVID-19 positives, evaluated the *ACE1* rs4646994 association with COVID-19 susceptibility (Table 1). Significant heterogeneity was observed for all genetic models with no significant association under the random model (Figure 2). Similar findings were detected in the meta-analysis of the *ACE1* rs4646994 variant with asymptomatic presentation (Table 2), indicating no significant effect pooled from three studies (Figure 3). We observed high heterogeneity in the sampling places and reported ethnic backgrounds.

**TABLE 1:** Association studies of *ACE1* rs4646994 (Alu 287 pb) with coronavirus disease 2019 (COVID-19) susceptibility included in the systematic review.

*Year*	*Author*	*Sample*					*Control*		*Case*	
		*Date*	*Place*	*Ethnic background*	*Size*	*Male n(%)*	*n*	*Criteria*	*n*	*Criteria*
2020	Gòmez	—	Spain	Caucasian (Asturias)	740	373 (0.50)	536	Healthy population	204	COVID-19 positive
2021	Akbari	2020	Iran	—	182	105 (0.57)	91	Unaffected individuals without a history of exposure to COVID-19 cases	91	COVID-19 positive
	Aladag	May/2020	Turkey	—	412	—	300	General population	112	COVID-19 positive
	Annunziata	March–April/20	Italy	Southern Italians	39	—	19	Healthy subjects	20	COVID-19 positive
	Hubacek	March–June/2020	Czech Republic	—	2,989	−(0.54)	2,579	General population	408	COVID-19 positive
	Kouhpayeh	May–September/2020	Iran	—	520	276 (0.55)	258	Healthy subjects with negative PCR and clinical diagnostic criteria	244	COVID-19 positive
	Mahmood	October–December/2020	Iraq	—	195	−(0.50)	96	Healthy subjects with negative serological test	99	COVID-19 positive
	Mir	September/2020–April/2021	Saudi Arabia	—	267	185 (0.69)	150	Healthy subjects	117	COVID-19 positive
	Möhlendick	March–September/2020	Germany	—	550	323 (0.59)	253	Patients with COVID-19 symptoms with negative PCR	297	COVID-19 positive
	Saad	—	Lebanon	Lebanese	387	195 (0.50)	155	Participants with negative PCR	232	COVID-19 positive
2022	Gong	January–March/2020	China	—	862	—	441	Healthy subjects	421	COVID-19 positive
	Papadopoulou	March–June/2020	Greece	Caucasian (Greek)	389	—	316	Blood product donors and volunteer healthcare workers	73	COVID-19 positive

**TABLE 2:** Association studies of *ACE1* rs4646994 (Alu 287 pb) with COVID-19 prognosis included in the systematic review.

*Phenotype*	*Year*	*Author*	*Sample*					*Control*		*Case*	
			*Date*	*Place*	*Ethnic background*	*Size*	*Male n(%)*	*n*	*Criteria*	*n*	*Criteria*
Asymptomatic × symptomatic	2021	Cafiero	—	Italy	—	104	58 (0.56)	50	Asymptomatic	54	Symptomatic (x-ray imaging)
		Hubacek	March–June/2020	Czech Republic	—	408	−(0.55)	163	Asymptomatic	245	Symptomatic (no hospitalization)
		Gunal	April–July/2020	Turkey	—	60	—	30	Asymptomatic	30	Severe (RR ≥30/min; SpO_2_ ≤93%; PaO_2_/FiO_2_ ≤300 mmHg; mechanical ventilation or ICU)
Nonsevere × severe	2020	Gòmez	—	Spain	Caucasian (Asturias)	204	125 (0.61)	137	Mild (hospitalized, nonsevere)	67	Severe (hospitalized, mechanical ventilation and/or ICU)
	2021	Akbari	2020	Iran	—	91	53 (0.58)	54	Hospitalized, non-ICU	37	Hospitalized, ICU
		Aladag	May/2020	Turkey	—	65	-	53	Nonsevere	12	Severe (fever or suspected respiratory infection, plus one of the following: RR >30/min; severe respiratory distress; or SpO_2_ ≤93%)
		Çelik	—	Turkey	—	154	78 (0.50)	119	Mild (outpatients) and moderate (hospitalized nonsevere)	35	Severe (RR ≥30/min; SpO_2_ ≤93%; PaO_2_/FiO_2_ ≤300 mmHg; mechanical ventilation or ICU)
		Gunal	April–July/2020	Turkey	—	90	-	60	Asymptomatic and mild	30	Severe (RR ≥30/min; SpO_2_ ≤93%; PaO_2_/FiO_2_ ≤300 mmHg; mechanical ventilation or ICU)
		Kouhpayeh	May–September/2020	Iran	—	258	144 (0.56)	106	Nonsevere	152	Severe (fever or suspected respiratory infection, plus one of the following: RR >30/min; severe respiratory distress; or SpO_2_ ≤93%)
		Mahmood	October–December/2020	Iraq	—	99	−(0.51)	68	Mild (with symptoms of pneumonia and no signs of severe pneumonia)	31	Severe (severe respiratory distress, RR ≥30 breaths/min or SpO_2_ ≤ 93%)
		Möhlendick	March–September/2020	Germany	—	251	176 (0.59)	207	Mild and hospitalized (non-ICU)	44	Severe (hospitalized, mechanical ventilation and/or ICU)
		Saad	-	Lebanon	Lebanese	223	123 (0.55)	162	Mild and moderate	61	Severe (lung infiltrates on chest x-ray or CT scan and SpO_2_ <94% who required hospitalization with essential oxygen therapy or mechanical ventilation)
		Verma	August–September/2020	India	India	269	174 (0.65)	149	Mild (RR <24/min, SpO_2_ >94%)	120	Severe (pneumonia with RR > 30/min; severe respiratory distress; or SpO_2_ ≤93%)
	2022	Gong	January–March/2020	China	—	421	—	318	Mild and moderate	103	Severe
		Papadopoulou	March–June/2020	Greece	Caucasian (Greek)	81	43 (0.53)	29	Mild and moderate (with symptoms of pneumonia and no signs of severe pneumonia)	52	Severe or critical (fever or suspected respiratory infection, plus one of the following: RR >30/min; severe respiratory distress; or SpO_2_ ≤93%)
Alive × dead	2021	Mir	September/2020–April/2021	Saudi Arabia	—	117	85 (0.73)	74	Alive	43	Dead
		Möhlendick	March–September/2020	Germany	—	297	176 (0.59)	251	Mild, hospitalized (non-ICU) and severe	46	Dead

Note. RR, respiratory rate; ICU, intensive care unit; SpO_2_, oxygen saturation; PaO_2_/FiO_2_, arterial oxygen pressure/fraction of inspired oxygen; CT, computerized tomography.

**FIGURE 4:**
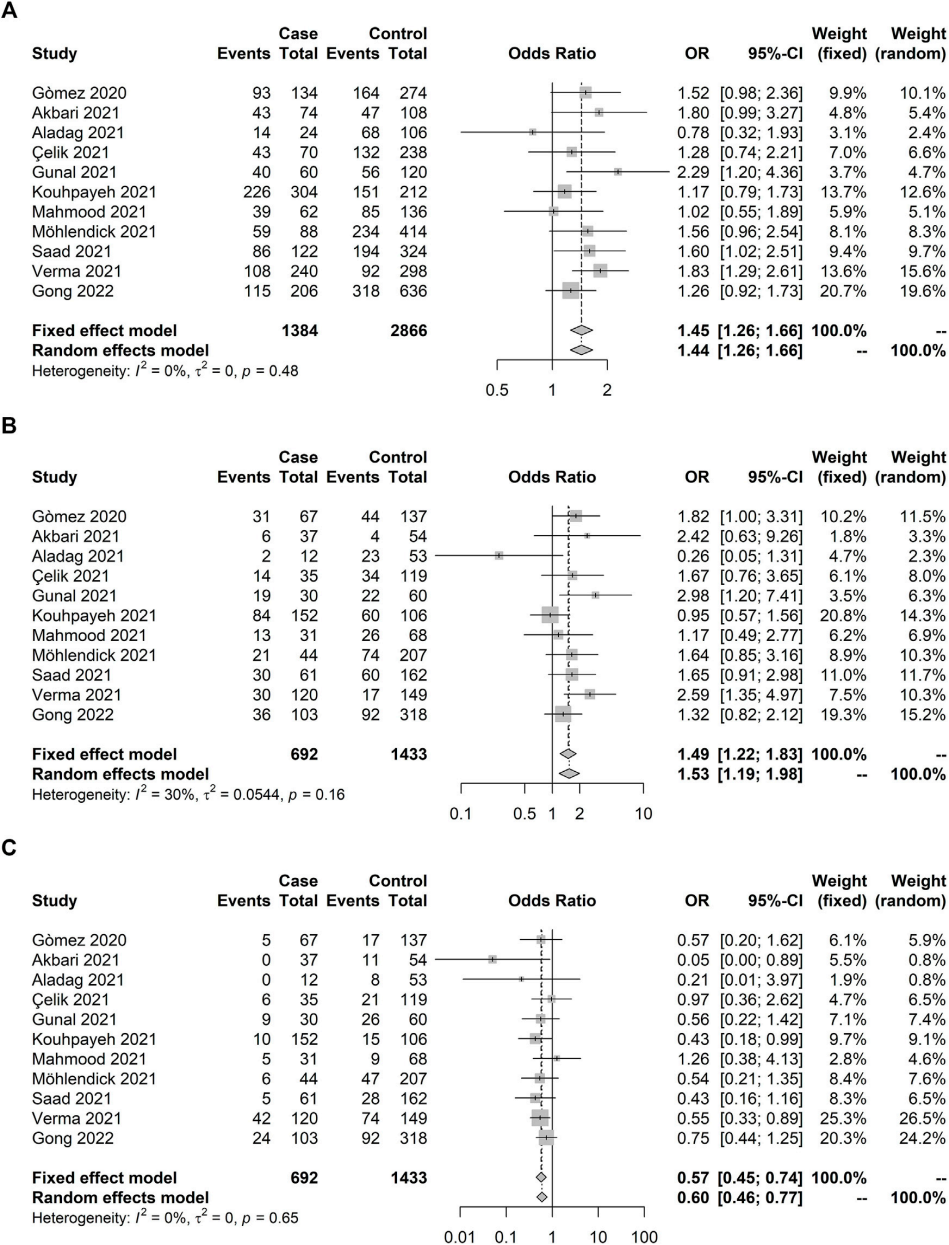
Forest plot illustrating *ACE1* rs4646994 (Alu 287 pb) association with COVID-19 severity (severe × others). Significant allelic and genotypic effects were observed. Case and control definitions are presented in Table 2. **(A)** D-allele model. D-allele was associated with increased risk of COVID-19 severity. **(B)** D recessive model. D/D genotype carriers showed increased odds to manifest severe COVID-19 compared with D/I and I/I carriers combined **(C)** I recessive model. I/I genotype carriers showed decreased odds to present severe COVID-19 compared with D/I and D/D carriers combined.

We were able to conduct a meta-analysis investigating whether *ACE1* rs4646994 polymorphism could predict COVID-19 severity. Eleven studies were included reaching a total of 692 individuals with severe COVID-19 and 1,433 with nonsevere manifestation (Table 2). The allelic association was observed with increased odds for deletion (D) allele compared with I-allele (pooled OR: 1.45; 95% CI: 1.26–1.66) (Figure 4A). Homozygous deletion (D/D) carriers showed 49% increased odds to present severe COVID-19 compared with heterozygous (D/I) and homozygous insertion allele (I/I) carriers combined (pooled OR: 1.49, 95% CI: 1.22–1.83) (Figure 4B). On the other hand, the I/I genotype was protective against severe COVID-19 (pooled OR: 0.57, 95% CI: 0.45–0.74) (Figure 4C).

**FIGURE 5:**
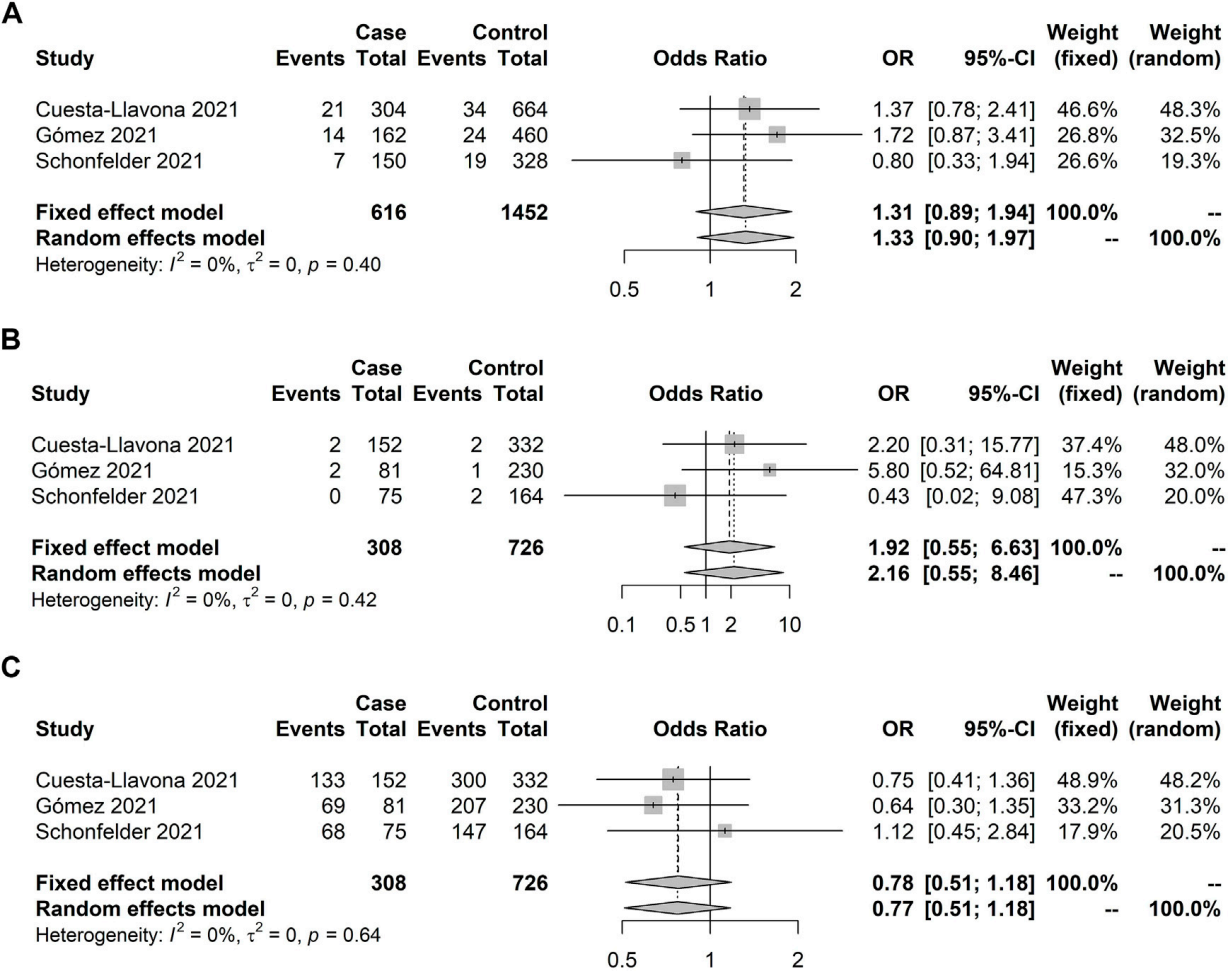
Forest plot illustrating *IFITM3* rs12252 association with severity (non-ICU × ICU). No significant results were observed. Case and control definitions are presented in Table 3. **(A)** C allele association. **(B)** C recessive model. **(C)** T recessive model.

**FIGURE 6:**
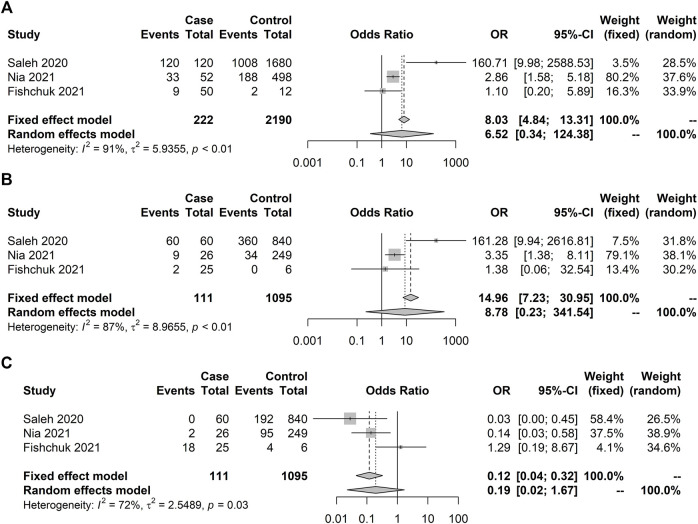
Forest plot illustrating *TNF-α* rs1800629 association with death (alive × dead). No significant results were observed under the random model. Case and control definitions are presented in Table 5. **(A)** C allele association. **(B)** C recessive model. **(C)** T recessive model.

The *IFITM3* rs12252 meta-analysis with severity included three studies totaling 308 individuals admitted to an intensive care unit and 726 who were not admitted (Table 3). No significant association was observed under any genetic model (Figure 5). Meta-analysis for other outcomes with the *IFITM3* rs12252 could not be conducted. The *TNF-α* rs1800629 association with death was analyzed in three studies (Table 4), including 111 subjects who died and 1,095 survivors. No significant association was observed under the random-effect models (Figure 6). *FURIN* (Table 5) genetic variants had less than three studies; therefore, no meta-analyses were carried out.

**TABLE 3:** Association studies of *IFITM3* rs12252 with COVID-19 prognosis included in the systematic review.

*Phenotype*	*Year*	*Author*	*Sample*					*Control*		*Case*	
			*Date*	*Place*	*Ethnic background*	*Size*	*Male n(%)*	*n*	*Criteria*	*n*	*Criteria*
Non-ICU × ICU	2021	Alghamdi	—	Saudi Arabia	Saudi	376	112 (0.56)	210	Hospitalized, non-ICU	166	Hospitalized, ICU
	2021	Cuesta-Llavona	March–December/2020	Spain	Caucasian (Asturias)	484	276 (0.57)	332	Hospitalized, non-ICU	152	Hospitalized, ICU
	2021	Gómez	March–August/2020	Not informed	Caucasian (Asturias)	311	174 (0.56)	230	Hospitalized, non-ICU	81	Hospitalized, ICU
	2021	Schonfelder	March–September/2020	Germany	Caucasian	239	141 (0.59)	164	Outpatients and hospitalized (non-ICU)	75	Hospitalized (ICU or mechanical ventilation) or dead
Alive × dead	2021	Alghamdi	—	Saudi Arabia	Saudi	861	—	784	Alive	77	Dead
	2021	Cuesta-Llavona	March–December/2020	Spain	Caucasian (Asturias)	484	276 (0.57)	114	Alive	38	Dead
Other	2020	Zhang	January–February/2020	China	—	80	33 (0.41)	56	Mild (hospitalized with fever, respiratory symptoms, and pneumonia seen with imaging)	24	Severe (RR ≥30/min; SpO_2_ ≤93%; PaO_2_/FiO_2_ ≤300 mmHg; mechanical ventilation or ICU)
	2021	Alghamdi	—	Saudi Arabia	Saudi	861	—	457	Nonhospitalized	374	Hospitalized

Note. RR, respiratory rate; ICU, intensive care unit; SpO_2_, oxygen saturation; PaO_2_/FiO_2_, arterial oxygen pressure/fraction of inspired oxygen.

**TABLE 4:** Association studies of *TNF-α* rs1800629 gene with COVID-19 prognosis or susceptibility included in the systematic review.

*Year*	*Author*	*Sample*					*Control*		*Case*	
		*Date*	*Place*	*Ethnic background*	*Size*	*Male n(%)*	*n*	*Criteria*	*n*	*Criteria*
2020	Saleh	April—July/2020	Egypt	—	1,084	600 (0.56)	184	Health care workers	900	COVID-19 positive
					900	-	444	Mild	456	Severe
					900	504 (0.56)	840	Alive	60	Dead
2021	Nia	June/2020—January/2021	Iran	—	550	234 (0.43)	275	COVID-19 negative	275	Hospitalized
					275	112 (0.41)	96	Nonsevere	179	Severe
					275	-	249	Alive	26	Dead
2021	Fishchuk	April–June/2020	Ukraine	—	31	16 (0.50)	25	Alive	6	Dead

**TABLE 5:** Association studies of *FURIN* gene with COVID-19 prognosis or susceptibility included in the systematic review.

*Year*	*Author*	*Sample*					*Control*		*Case*	
		*Date*	*Place*	*Ethnic background*	*Size*	*Male n(%)*	*n*	*Criteria*	*n*	*Criteria*
2020	Latini	Mar—May/2020	Italy	—	—	—	—	Severe (respiratory impairment, requiring noninvasive ventilation)	—	Extremely severe (requiring invasive ventilation and ICU)
					131	82 (0.63)	—	Asymptomatic	—	Severe and extremely severe
2021	Torre-Fuentes	—	Spain	—	120	-	113	COVID-19 negative	7	COVID-19 positive

## Discussion

We conducted a systematic review followed by meta-analysis including studies covering genetic association of COVID-19 susceptibility or prognosis with *IFITM3*, *FURIN*, *ACE1*, and *TNF-α* variants. Four studies included in the meta-analyses did not report the sample collection date, which is of particular interest in COVID-19 studies due to the emergence of variants of concern (VOCs) in the last part of 2020 (Konings et al., 2021). Some VOCs have been associated with higher viral load, worse prognosis, and lethality (Davies et al., 2021; Faria et al., 2021), thus, confounding factors when evaluating genetic effects. Age can also be a confounding factor for COVID-19 association analysis (Fernández Villalobos et al., 2021). Most studies failed to conduct age-corrected estimation or even describe age separately for case and control groups. The same trend was observed for comorbidities (data now shown).

Ancestrality could also contribute to COVID-19 outcomes. Several studies do not present the ethnic background or, at least, the place of birth of the included subjects. Although heterogeneity was seen in parameters associated with ancestrality, the literature fails on genetic background diversity, an issue already raised for genomic data before (Popejoy and Fullerton, 2016). Another literature issue that needs attention is the selective reporting biases leading to the more likely publication of positive findings (Munafò et al., 2009; Sagoo et al., 2009).

We did not find an association of *IFITM3* rs12252 with Covid-19 severity. Our results corroborate the most extensive association study published to date since no significance was reported on any of chromosome 11 loci (Niemi et al., 2021). However, the second evaluated polymorphism, the *ACE1* rs4646994, showed significant effects with homozygous D carriers presenting higher odds of developing severe COVID-19. Several hits on the large arm of chromosome 17 have been previously reported (Niemi et al., 2021), although their genomic location is too far to hypothesize linkage disequilibrium. It is important to note that genome-wide data may find hits on loci that not necessarily are the ones harboring the causative variants because of its experimental design (Spencer et al., 2009). Furthermore, candidate-gene, whole-exome or whole-genome sequencing studies are more suitable in exploring large indel variants.

The *ACE1* rs4646994 has been associated with several clinical phenotypes, including COVID-19 (Castellon and Hamdi, 2007; Li et al., 2021). Most previous findings report associations with COVID-19 outcomes on a population level, indicating high variability on allelic frequencies across different populations (Delanghe et al., 2020; Pati et al., 2020; Yamamoto et al., 2020). On a molecular level, expression results indicate increased levels of ACE1 in D-allele carriers (Suehiro et al., 2004) with increased angiotensin II production (Hamdi and Castellon, 2004) and decreased ACE2 protein levels in lung tissue, thereby potentially affecting infectivity by SARS-CoV-2 (Jacobs et al., 2021). Our group has previously indicated that lower ACE2 levels may increase the risk of COVID-19 respiratory distress (Rossi et al., 2021). Although there is a robust biological hypothesis linking *ACE1* rs4646994 with COVID-19, further reports are needed to understand better whether *ACE1* variants could contribute to COVID-19 severity. Moreover, studies are still required to adequately evaluate *IFITM3*, *FURIN*, and *TNF-α* genetic variants’ role in COVID-19 susceptibility and outcomes.

## Data Availability

The original contributions presented in the study are included in the article/Supplementary Material, further inquiries can be directed to the corresponding author.
