# Supplementary material for: IFITM3, FURIN, ACE1, and TNF-α Genetic Association With COVID-19 Outcomes: Systematic Review and Meta-Analysis
Source: Front Genet. 2022 Apr 1;13:775246. doi: 10.3389/fgene.2022.775246 (PMC9010674; doi:10.3389/fgene.2022.775246)
Supplement: Supplementary file 1 [file DataSheet1.pdf]

IFITM3, _FURIN_, _ACE1_ and _TNF- α_ genetic association with Covid-19 outcomes: systematic review and meta-analysis

João Locke Ferreira de Araújo[1]; Diego Menezes [1];
Renato Santana de Aguiar[1]; Renan Pedra de Souza [1]

[1] Grupo de Pesquisa em Bioestatística e Epidemiologia Molecular; Laboratório de Biologia Integrativa; Programa de Pós Graduação em Genética; Departamento de Genética, Ecologia e Evolução; Instituto de Ciências Biológicas; Universidade Federal de Minas Gerais

Running Title: *IFITM3*, *FURIN*, *ACE1* and *TNF-α* SNPs and Covid-19

Corresponding author
Renan P. Souza (renanrps@ufmg.br)
Universidade Federal de Minas Gerais
Av. Antônio Carlos, 6627 ICB - Pampulha
31270901 - Belo Horizonte - Minas Gerais - Brazil
Phone: +553134092570

Supplementary material 1

Search strategy

***IFITM3* gene**

(SARS-Cov-2[Title/Abstract] OR covid-19[Title/Abstract] OR 2019-nCov [Title/Abstract] OR SARS-CoV2 [Title/Abstract])

AND

(allele[Title/Abstract] OR genotype[Title/Abstract] OR "genes"[MeSH Terms] OR "polymorphism, genetic"[MeSH Terms] OR "genetic association studies"[MeSH Terms] OR "genotype"[MeSH Terms] OR "genetic predisposition to disease"[MeSH Terms] OR "genetic variation"[MeSH Terms] OR "alleles"[Title/Abstract] OR "genes"[Title/Abstract] OR "polymorphism, genetic"[Title/Abstract] OR "genetic association studies"[Title/Abstract] OR "genotype"[Title/Abstract] OR "genetic predisposition to disease"[Title/Abstract] OR "genetic variation"[Title/Abstract] OR "alleles"[Title/Abstract])

AND

(IFITM3 [Title/Abstract] OR "Interferon Induced Transmembrane Protein 3" [Title/Abstract] OR DSPA2b [Title/Abstract] OR "Interferon-Induced Transmembrane Protein 3" [Title/Abstract] OR "Interferon-Inducible Protein 1-8U" [Title/Abstract] OR 1-8U [Title/Abstract] OR "Interferon Induced Transmembrane Protein 3 (1-8U)" [Title/Abstract] OR IP15 [Title/Abstract])

***FURIN* gene**

(SARS-Cov-2[Title/Abstract] OR covid-19[Title/Abstract] OR 2019-nCov [Title/Abstract] OR SARS-CoV2 [Title/Abstract])

AND

(allele[Title/Abstract] OR genotype[Title/Abstract] OR "genes"[MeSH Terms] OR "polymorphism, genetic"[MeSH Terms] OR "genetic association studies"[MeSH Terms] OR "genotype"[MeSH Terms] OR "genetic predisposition to disease"[MeSH Terms] OR "genetic variation"[MeSH Terms] OR "alleles"[Title/Abstract] OR "genes"[Title/Abstract] OR "polymorphism, genetic"[Title/Abstract] OR "genetic association studies"[Title/Abstract] OR "genotype"[Title/Abstract] OR "genetic predisposition to disease"[Title/Abstract] OR "genetic variation"[Title/Abstract] OR "alleles"[Title/Abstract])

AND

(Furin [Title/Abstract] OR "Furin, Paired Basic Amino Acid Cleaving Enzyme" [Title/Abstract] OR "Paired Basic Amino Acid Residue-Cleaving Enzyme" [Title/Abstract] OR "Proprotein Convertase Subtilisin/Kexin 3" [Title/Abstract] OR "Subtilisin-Like Proprotein Convertase 1" [Title/Abstract] OR "EC 3.4.21.75" [Title/Abstract] OR PCSK3 [Title/Abstract] OR PACE [Title/Abstract] OR SPC1 [Title/Abstract])

***ACE1* gene**

(SARS-Cov-2[Title/Abstract] OR covid-19[Title/Abstract] OR 2019-nCov [Title/Abstract] OR SARS-CoV2 [Title/Abstract])

AND

(allele[Title/Abstract] OR genotype[Title/Abstract] OR "genes"[MeSH Terms] OR "polymorphism, genetic"[MeSH Terms] OR "genetic association studies"[MeSH Terms] OR "genotype"[MeSH Terms] OR "genetic predisposition to disease"[MeSH Terms] OR "genetic variation"[MeSH Terms] OR "alleles"[Title/Abstract] OR "genes"[Title/Abstract] OR "polymorphism, genetic"[Title/Abstract] OR "genetic association studies"[Title/Abstract] OR "genotype"[Title/Abstract] OR "genetic predisposition to disease"[Title/Abstract] OR "genetic variation"[Title/Abstract] OR "alleles"[Title/Abstract])

AND

(ACE1 [Title/Abstract] OR “Angiotensin I Converting Enzyme” [Title/Abstract] OR “Angiotensin I Converting Enzyme (Peptidyl-Dipeptidase A) 1” [Title/Abstract] OR “Angiotensin-Converting Enzyme” [Title/Abstract] OR “Dipeptidyl Carboxypeptidase I” [Title/Abstract] OR “CD143 Antigen” [Title/Abstract] OR “Kininase II” [Title/Abstract] OR CD143 [Title/Abstract] OR DCP1 [Title/Abstract] OR DCP [Title/Abstract] OR “Dipeptidyl Carboxypeptidase 1” [Title/Abstract] OR “Peptidyl-Dipeptidase A” [Title/Abstract] OR Carboxycathepsin [Title/Abstract] OR “Peptidase P” [Title/Abstract] OR “EC 3.4.15.1” [Title/Abstract] OR “EC 3.2.1” [Title/Abstract] OR ACE [Title/Abstract] OR ECA [Title/Abstract])

***TNF-α* gene**

(SARS-Cov-2[Title/Abstract] OR covid-19[Title/Abstract] OR 2019-nCov [Title/Abstract] OR SARS-CoV2 [Title/Abstract])

AND

(allele[Title/Abstract] OR genotype[Title/Abstract] OR "genes"[MeSH Terms] OR "polymorphism, genetic"[MeSH Terms] OR "genetic association studies"[MeSH Terms] OR "genotype"[MeSH Terms] OR "genetic predisposition to disease"[MeSH Terms] OR "genetic variation"[MeSH Terms] OR "alleles"[Title/Abstract] OR "genes"[Title/Abstract] OR "polymorphism, genetic"[Title/Abstract] OR "genetic association studies"[Title/Abstract] OR "genotype"[Title/Abstract] OR "genetic predisposition to disease"[Title/Abstract] OR "genetic variation"[Title/Abstract] OR "alleles"[Title/Abstract])

AND

(TNF [Title/Abstract] OR “Tumor Necrosis Factor” [Title/Abstract] OR “TNF-Alpha” [Title/Abstract] OR TNFSF2 [Title/Abstract] OR “Tumor Necrosis Factor Ligand Superfamily Member 2 ” [Title/Abstract] OR TNF-A [Title/Abstract] OR TNFA [Title/Abstract] OR DIF [Title/Abstract] OR “Tumor Necrosis Factor (TNF Superfamily, Member 2)” [Title/Abstract] OR “Tumor Necrosis Factor Ligand 1F “ [Title/Abstract] OR “Tumor Necrosis Factor-Alpha” [Title/Abstract] OR “TNF Superfamily, Member 2” [Title/Abstract] OR “TNF, Macrophage-Derived” [Title/Abstract] OR “TNF, Monocyte-Derived” [Title/Abstract] OR “APC1 Protein ” [Title/Abstract] OR Cachectin [Title/Abstract] OR TNLG1F [Title/Abstract])

Table S1: Q-Genie quality results of the included reports in the metanalysis.

Authors	Gene	Question 1	Question 2	Question 3	Question 4	Question 5	Question 6	Question 7	Question 8	Question 9	Question 10	Question 11	Final	Concept
Zhang et al., 2020	*IFTIM3*	5.00	3.67	3.00	1.00	1.00	3.00	1.00	7.00	5.00	1.00	7.00	37.67	Moderate
Gómez et al., 2021	*IFTIM3*	7.00	3.00	2.50	5.00	1.00	5.00	2.00	3.50	5.00	3.00	3.00	40.00	Moderate
Alghamdi et al., 2021	*IFTIM3*	7.00	1.67	1.50	3.00	1.00	7.00	4.00	7.00	5.00	1.00	3.00	41.17	Moderate
Schonfelder at al., 2021	*IFTIM3*	7.00	4.00	3.25	3.00	1.00	5.00	2.00	5.00	6.33	3.00	7.00	46.58	Good
Cuesta-Llavona et al 2021	*IFTIM3*	7.00	5.00	4.00	2.67	1.00	7.00	7.00	6.00	7.00	3.00	7.00	56.67	Good
Gòmez et al., 2020	*ACE1*	7.00	4.33	3.50	2.20	1.00	7.00	2.00	4.50	7.00	1.00	7.00	46.53	Good
Verma et al., 2021	*ACE1*	5.00	3.67	3.00	3.40	1.00	7.00	3.00	5.50	7.00	1.00	3.00	42.57	Moderate
Gunal et al., 2021	*ACE1*	7.00	3.00	2.50	2.20	1.00	7.00	4.00	7.00	7.00	1.00	5.00	46.70	Good
Çelik et al., 2021	*ACE1*	7.00	3.00	2.50	2.20	1.00	5.00	1.00	7.00	5.67	3.00	7.00	44.37	Good
Akbari et al., 2021	*ACE1*	7.00	3.67	2.50	4.67	1.00	5.00	3.50	6.50	5.67	2.00	5.00	46.50	Good
Aladag et al., 2021	*ACE1*	7.00	3.67	2.50	4.00	1.00	7.00	4.00	6.00	5.00	3.00	7.00	50.17	Good
Mir et al., 2021	*ACE1*	7.00	4.33	2.00	6.00	1.00	7.00	4.00	6.50	5.00	3.00	4.00	49.83	Good
Annunziata et al 2021	*ACE1*	7.00	1.00	2.50	1.00	1.00	7.00	1.50	2.50	3.00	1.00	1.00	28.50	Poor
Cafiero et al 2021	*ACE1*	7.00	1.67	1.00	1.67	1.00	1.00	2.00	6.00	4.33	3.00	7.00	35.67	Moderate
Kouhpayeh et al 2021	*ACE1*	7.00	3.67	3.00	5.00	1.00	3.00	4.00	6.50	5.67	3.00	4.00	45.83	Good
Mahmood et al 2022	*ACE1*	7.00	3.67	3.00	2.67	1.00	3.00	6.50	6.00	5.67	1.00	5.00	44.50	Moderate
Möhlendick et al 2021	*ACE1*	7.00	4.33	2.50	2.33	1.00	7.00	4.00	5.50	7.00	3.00	7.00	50.67	Good
Papadopoulou et al 2021	*ACE1*	7.00	3.00	4.00	4.00	1.00	5.00	4.00	6.00	5.67	3.00	5.00	47.67	Good
Saad et al 2021	*ACE1*	7.00	3.00	1.00	4.00	1.00	7.00	4.00	7.00	7.00	5.00	7.00	53.00	Good
Gong et al., 2022	*ACE1*	3.00	5.00	5.50	2.33	1.00	7.00	4.00	6.50	3.00	3.00	3.00	43.33	Moderate
Hubacek et al., 2021	*ACE1*	7.00	1.00	1.50	3.00	1.00	1.00	4.00	2.50	1.00	1.00	7.00	30.00	Poor
